# Telehealth Services for Substance Use Disorders During the COVID-19 Pandemic: Longitudinal Assessment of Intensive Outpatient Programming and Data Collection Practices

**DOI:** 10.2196/36263

**Published:** 2022-03-14

**Authors:** Kate Gliske, Justine W Welsh, Jacqueline E Braughton, Lance A Waller, Quyen M Ngo

**Affiliations:** 1 Butler Center for Research Hazelden Betty Ford Foundation Center City, MN United States; 2 Department of Psychiatry and Behavioral Services Emory University School of Medicine Emory University Atlanta, GA United States; 3 Department of Biostatistics and Bioinformatics Emory University Rollins School of Public Health Emory University Atlanta, GA United States

**Keywords:** telehealth, substance use disorder, COVID-19, substance use treatment, feasibility study, routine outcome monitoring data, mental health, addiction, digital health, telemedicine, outpatient program, virtual health, addiction treatment, virtual care, patient outcomes

## Abstract

**Background:**

The onset of the COVID-19 pandemic necessitated the rapid transition of many types of substance use disorder (SUD) treatments to telehealth formats, despite limited information about what makes treatment effective in this novel format.

**Objective:**

This study aims to examine the feasibility and effectiveness of virtual intensive outpatient programming (IOP) treatment for SUD in the context of a global pandemic, while considering the unique challenges posed to data collection during an unprecedented public health crisis.

**Methods:**

The study is based on a longitudinal study with a baseline sample of 3642 patients who enrolled in intensive outpatient addiction treatment (in-person, hybrid, or virtual care) from January 2020 to March 2021 at a large substance use treatment center in the United States. The analytical sample consisted of patients who completed the 3-month postdischarge outcome survey as part of routine outcome monitoring (n=1060, 29.1% response rate).

**Results:**

No significant differences were detected by delivery format in continuous abstinence (*χ*^2^_2_=0.4, *P*=.81), overall quality of life (*F*_2,826_=2.06, *P*=.13), financial well-being (*F*_2,767_=2.30, *P*=.10), psychological well-being (*F*_2,918_=0.72, *P*=.49), and confidence in one’s ability to stay sober (*F*_2,941_=0.21, *P*=.81). Individuals in hybrid programming were more likely to report a higher level of general health than those in virtual IOP (*F*_2,917_=4.19, *P*=.01).

**Conclusions:**

Virtual outpatient care for the treatment of SUD is a feasible alternative to in-person-only programming, leading to similar self-reported outcomes at 3 months postdischarge. Given the many obstacles presented throughout data collection during a pandemic, further research is needed to better understand under what conditions telehealth is an acceptable alternative to in-person care.

## Introduction

Substance use disorder (SUD) is a chronic relapsing disease associated with numerous psychosocial harms and health sequalae. Addiction was a leading global cause of disability and death prior to the COVID-19 pandemic [1], which has since disproportionately impacted individuals suffering from SUD. Recent studies indicate that individuals with SUD may be more susceptible to severe disease and have higher rates of mortality and postvaccination breakthrough infections [2-4]. Isolation, uncertainty, and financial instability have also compounded substance use and the challenges of early recovery [5-7]. These vulnerabilities have reinforced the critical need for ongoing and safe access to treatment throughout the course of the pandemic through virtual services.

Prior to the COVID-19 pandemic, virtual services showed promise but were slow to develop. Early applications have shown promise as a means of preventing premature dropout from SUD treatment [8]. The pandemic rapidly accelerated the implementation of telehealth services for mental health and substance use treatment [9], and both providers and participants have viewed these types of services favorably [10,11]. Unfortunately, little is known about the actual efficacy or effectiveness of individual SUD treatment in telehealth treatment settings [12-14], and even less about traditional group treatment formats [15].

The onset of the pandemic became a catalyst for addiction treatment programs to quickly pivot to provision of services through telehealth formats despite limited data to guide their delivery. Change was facilitated by paradigm shifts in federal, state, and local policies and in organizational and provider practices [16,17]. Although these policies allowed for the continuity of care through available technology, stakeholders within the addiction field are now facing decisions on which elements of policies and programs to sustain, adapt, or discontinue. Continuation of these policies is dependent upon rigorous assessment of clinical data to define the new standard of SUD treatment through virtual platforms. Unfortunately, the pandemic had a devastating impact on research, with ongoing disruptions to recruitment and study progress, as well as a dramatic reduction in survey participation and response rates across many fields of study [18-20].

There is still a significant need for research related to the application and assessment of telehealth for SUD. Unfortunately, best practices for patient outcome collection for SUD treatment in mixed settings have yet to be established. In this paper, we describe how the COVID-19 pandemic presented a novel opportunity to bridge the gap and assess the effectiveness of a virtual intensive outpatient programming (IOP) for substance use treatment through the examination of short-term postprogram outcomes of adults who received IOP services through different delivery formats at the largest SUD treatment provider in the United States.

## Methods

### Study Design and Population

The Hazelden Betty Ford Foundation (HBFF) is the largest national provider of addiction services in the United States. The HBFF utilizes evidence-based practices through a multidisciplinary and integrated approach to addiction treatment across varying levels of care. In 2019, the HBFF piloted a single virtual intensive outpatient group with planned expansion of virtual services in 2020, as informed by routine outcome monitoring (ROM) data. The HBFF has an established infrastructure and process for collection of ROM data that has been used and refined since 1974. ROM data provide an understanding of real-world conditions, offering applied generalizability to community-based treatment settings where the majority of care is provided [21,22]. These data can be designed as a feedback loop, intended to quickly translate findings into treatment implementation [23,24]. In a rapidly evolving global pandemic, this type of real-world feedback is invaluable to informing the refinement of virtual treatment, despite potentially lower response rates than a formal randomized controlled trial [22].

This study presents 3-month findings (n=1060, 29.1%) from a 12-month longitudinal assessment of patients, 18 years and older, who were discharged from IOP between January 2020 and March 2021 (N=3642). Patients were separated by 3 distinct treatment delivery settings in response to pandemic changes: (1) in-person care only (n=957, 26.3%); (2) hybrid, in-person, and virtual care (n=541, 14.9%); and (3) virtual care only (n=2144, 58.9%).

### Ethics Approval

The study was reviewed and approved by Emory University’s Institutional Review Board (STUDY00001822) and was determined to have met the human research exemption since all data were collected within the context of the HBFF’s standard ROM practices.

### Data Collection Procedures

Trained research data collection specialists (RDCS) utilized a systematic and manualized process for data collection. Web-based surveys were automatically assigned to IOP patients at 1, 3, 6, 9, and 12 months postdischarge, with survey completion windows open for 30 days. Survey links were emailed to patients, with reminder prompts every 3 days for up to 2 weeks. Patients were contacted by an RDCS every 4-7 days to complete the survey over the phone or to encourage patients to complete it online if not initially completed. Patients were still prompted to provide responses if admitted to a different level of care.

### Impact of the Pandemic on Data Collection

**Figure 1:**
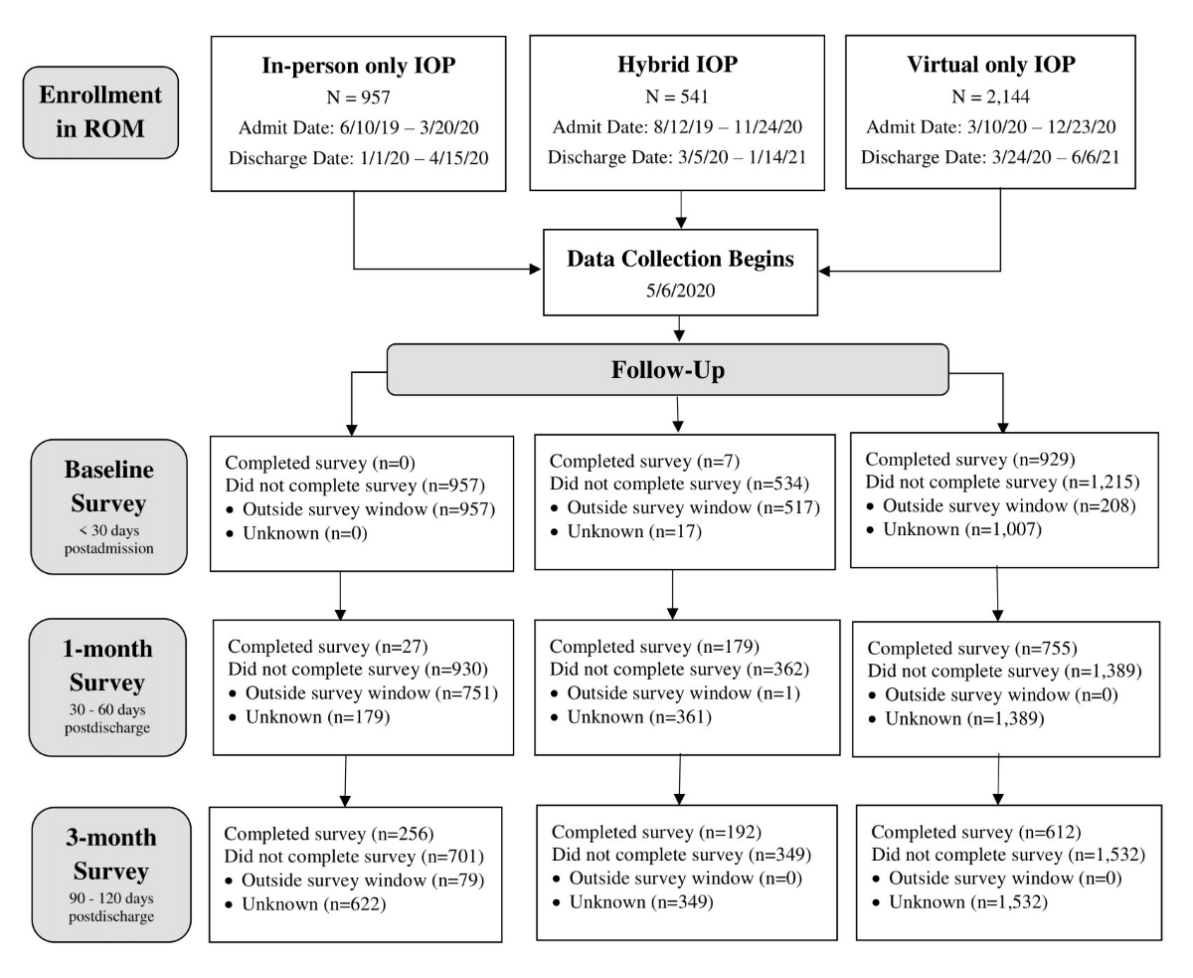
Data completeness from baseline to 3-month follow-up. IOP: intensive outpatient program; ROM: routine outcome monitoring.

In response to a notable decline in response rates at the beginning of the pandemic, RDCS spent over 1200 hours attempting to contact patients to complete the 3-month survey throughout the course of the virtual IOP study. The timing of data collection was also impacted. Virtual IOP began in March 2020, while data collection began in May 2020, largely due to reallocation of resources to facilitate the transition of direct care to virtual services. To retrospectively capture an in-person IOP comparison group, all patients discharged from an in-person IOP on or after January 1, 2020, were opted into receiving IOP outcome surveys. However, due to the 30-day survey windows, the majority of in-person and hybrid patients were excluded from completing the baseline survey at admission and the 1-month postdischarge survey, impacting response rates for those time points (Figure 1).

### Measures

Demographic information was collected from patient electronic medical records. The majority of the full sample of patients were White (n=3296, 91.3%) and male (n=2258, 62.0%), with a mean age of 39.1 (SD 13.5) years.

#### Outcome Measures

A variety of self-reported outcome measures were used to assess for health and well-being at 3-month follow-up. Continuous abstinence from drugs and alcohol (*abstinent since discharge* vs *relapsed*) during the follow-up period was assessed using a question from a modified Form 90 Alcohol Questionnaire (Form 90-AQ) [25], adapted to ask about the use of any substances and to include the specific time period for clarity: “Have you used any drugs or alcohol since your last survey on (last survey date)?” Compliance to prescribed anticraving medications (eg, buprenorphine, naltrexone, or acamprosate) was assessed with a single-item, binary question: “Have you taken your anticraving medication, as prescribed?” Peer support group engagement was measured by 1 item from the Alcoholics Anonymous (AA) Involvement Scale [26] and adapted to include a reference to other peer group support beyond AA: “About how often have you been attending 12-step/peer support/mutual aid group meetings since you were discharged?”. Participants rated their frequency of attendance on a 6-point scale: *daily*, *4 or more times per week*, *1-3 times per week*, *2-4 times per month*, *once a month or less*, and *never*.

Quality of life was measured using the 4-item self-reported Centers for Disease Control Healthy Days Survey [27,28]. An additional question assessing overall quality of life was also added: “How would you rate your overall quality of life?” Patients were asked to rate their overall quality of life and quality of general health using a 5-point Likert rating, from 1 (*poor*) to 5 (*excellent*), and indicated the number of days out of the previous 30 that they experienced poor mental or physical health. Higher numbers of unhealthy days indicated a lower quality of life. Psychological well-being was assessed by the summed composite of the 8-item Flourishing Scale [29]. For each item, patients rated their level of agreement on a 7-point Likert scale, from 1 (*strongly disagree*) to 7 (*strongly agree*). The scale yielded high internal consistency (*α*=.94). Financial well-being was measured using the 5-item Consumer Financial Protection Bureau (CFPB) Financial Well-Being Scale, with higher scores illustrating greater perceived financial well-being [30]. The CFPB Financial Well-Being Scale showed good internal consistency (*α*=.85). Patients’ confidence in their ability to stay sober was measured using an adapted form of the Brief Situational Confidence Questionnaire (BSCQ) to create a sobriety self-efficacy scale [31]. The 7-point Likert response categories were reworded to maintain consistency across the different scales, and the original BSCQ question 5, “I could probably go back to social drinking or other moderate drug use if I wanted to,” was removed as initial interitem correlations and *α* values indicated that this question did not adequately add to the measure of sobriety self-efficacy. After removal of question 5, the adapted scale of sobriety self-efficacy showed high internal consistency (*α*=.89).

### Statistical Analyses

Analyses were performed using IBM SPSS Statistics version 28 [32]*.* Data were examined using chi-square tests of independence and one-way ANOVAs to ascertain the relationship between IOP delivery setting and patient outcomes. Direct comparisons between the different settings of IOP were not indicated because the virtual IOP study was not prospectively designed and was instead reactively implemented as a result of the pandemic. This pandemic reality resulted in differences in the timing of care, akin to a cohort effect within a single year, where those in-person care reached the 3-month survey earlier in the pandemic (eg, May-July 2020), while those in virtual-only care completed the 3-month survey over a much longer period (eg, June 2020-June 2021).

## Results

### Sample Characteristics

Sample characteristics are reported by IOP modality in Table 1. Differences between IOP settings emerged in biological sex, age, and length-of-stay distributions. In comparison to in-person and virtual groups, the hybrid group members were more likely to be male (133/192 [69.3%] in the hybrid group vs 161/256 [62.9%] in the in-person group and 363/612 [59.3%] in the virtual group). The virtual group had a greater number of individuals aged from 45 to 64 years (293/612 [47.9%] in the virtual group vs 92/256 [35.9%] in the in-person group and 67/192 [34.9%] in the hybrid group). Individuals in the hybrid group had significantly longer lengths of stay (mean 74.67 [SD 41.78] days) than those who participated in in-person IOP (mean 53.88 [SD 34.79] days) or virtual IOP (mean 54.67 [SD 33.31] days). No significant differences were detected between formats by race, ethnicity, marital status, employment status, education level, whether a patient was discharged against staff/medical advice, use of insurance for services, or type or number of active SUD diagnoses.

Demographic and clinical characteristics of noncompleters of the 3-month survey were compared with those who completed the survey (Table 2). There were no significant differences in completer status in regard to biological sex, race, identification as Latinx, or the highest level of education attained. However, a few differences emerged in age, marital/relational status, and employment type. Completers of the 3-month survey were slightly older (mean age 42.26 [SD 12.93] years) than noncompleters (mean age 38.26 [SD 13.17] years). In addition, completers were more likely to be employed full-time (672/1060 [63.4%] vs 1400/2503 [55.9%]) and to be married/cohabiting (550/1060 [51.9%] vs 985/2503 [39.8%]).

A greater number of differences arose in regard to clinical characteristics. Those who completed the 3-month survey were less likely to have multiple active SUDs (336/1060 [31.7%] vs 1014/2503 [40.5%]) and to get discharged against staff/medical advice (107/1060 [10.1%] vs 531/2503 [21.2%]). Completers also showed a longer length of stay in IOP care (mean 58.10 [SD 36.16] days) than noncompleters (mean 49.57 [SD 38.73] days). There was no difference between those who stepped down into IOP from a higher level of programming within the HBFF (eg, residential vs day treatment).

**Table 1:** Baseline characteristics of 3-month outcomes survey respondents (N=1060).^a^

Characteristics			In-person only (N=256)		Hybrid (N=192)	Virtual only (N=612)		Overall (N=1060)
**Biological sex, n (%); *χ*^2^_2_=6.3, *P*=.04**								
	Male	161 (62.9)		133 (69.3)		363 (59.3)	657 (62.0)	
	Nonbinary	N/A^b^		N/A		N/A	N/A	
	Missing	N/A		N/A		N/A	0	
**Age (years), n (%);*χ*^2^_6_=19.5, *P*=.003**								
	18-25	34 (13.3)		33 (17.2)		64 (10.5)	131 (12.4)	
	26-44	120 (46.9)		86 (44.8)		241 (39.4)	447 (42.2)	
	45-64	92 (35.9)		67 (34.9)		293 (47.9)	452 (42.6)	
	65+	10 (3.9)		6 (3.1)		14 (2.3)	30 (2.8)	
	Missing	N/A		N/A		N/A	0	
**Race^c^, n (%); *χ*^2^_2_=11.3, *P*=.88**								
	American Indian or Alaska Native	2 (0.8)		1 (0.5)		6 (1.0)	9 (0.8)	
	Asian or Asian American	3 (1.2)		2 (1.0)		3 (0.5)	8 (0.8)	
	Black or African American	3 (1.2)		4 (2.1)		12 (2.0)	19 (1.8)	
	Native Hawaiian or other Pacific Islander	1 (0.4)		0		1 (0.2)	2 (0.2)	
	White	233 (91.0)		180 (93.8)		560 (91.5)	973 (91.8)	
	Biracial or multiracial (2+)	6 (2.3)		3 (1.6)		9 (1.5)	18 (1.7)	
	Other	7 (2.7)		2 (1.0)		13 (2.2)	22 (2.1)	
	Missing	1 (0.4)		0		8 (1.3)	9 (0.9)	
**Ethnicity, n (%); *χ*^2^_2_=2.3, *P*=.31**								
	Hispanic or Latinx or Spanish origin	16 (6.3)		6 (3.1)		29 (4.7)	51 (4.8)	
	Not Hispanic or Latinx or Spanish origin	232 (90.6)		180 (93.8)		551 (90.0)	963 (90.8)	
	Missing	8 (3.1)		6 (3.1)		32 (5.2)	46 (4.3)	
**Marital status^c^, n (%); *χ*^2^_4_=6.3, *P*=.18**								
	Single/never married	87 (34.0)		78 (40.6)		204 (33.3)	369 (34.8)	
	Cohabiting	4 (1.6)		3 (1.6)		11 (1.8)	18 (1.7)	
	Married/life partner	125 (48.8)		95 (49.5)		312 (50.9)	532 (50.1)	
	Married but separated	14 (5.5)		6 (3.1)		19 (3.1)	39 (3.7)	
	Divorced	21 (8.2)		9 (4.7)		57 (9.3)	87 (8.2)	
	Widowed	3 (1.2)		1 (0.5)		1 (0.2)	5 (0.5)	
	Missing	2 (0.8)		0		8 (1.3)	10 (0.9)	
**Employment status^c^, n (%); *χ*^2^_2_=1.9, *P*=.76**								
	Full-time employment/Self-employed	164 (64.1)		119 (62.0)		389 (63.6)	672 (63.4)	
	Part-time employment	12 (4.7)		4 (2.1)		21 (3.4)	38 (3.6)	
	Home and family manager	6 (2.3)		3 (1.6)		9 (1.5)	18 (1.7)	
	Student (full- or part-time) or retired	18 (7.0)		21 (11.0)		36 (5.9)	74 (7.0)	
	Unemployment, actively seeking a job	16 (6.3)		6 (3.1)		25 (4.1)	47 (4.4)	
	Unemployment, not seeking a job	36 (14.1)		36 (18.8)		113 (18.5)	185 (17.5)	
	Missing	4 (1.6)		3 (1.5)		19 (2.9)	26 (2.5)	
**Education level^c^, n (%); *χ*^2^_4_=8.4, *P*=.08**								
	Some high school or less, no diploma	1 (0.4)		3 (1.5)		6 (1.0)	10 (1.0)	
	High school diploma or equivalent (General Educational Development [GED])	17 (6.6)		23 (12.0)		66 (10.8)	106 (10.0)	
	Some college, no degree	31 (12.1)		34 (17.7)		73 (11.9)	138 (13.0)	
	Associate degree/vocational-technical studies	15 (5.9)		14 (7.3)		36 (5.9)	65 (6.1)	
	College graduate/bachelor’s degree	85 (33.2)		56 (29.2)		166 (27.1)	307 (29.0)	
	Graduate/professional degree	35 (13.7)		18 (9.4)		61 (10.0)	114 (10.8)	
	Missing	72 (28.1)		44 (22.9)		204 (33.4)	320 (30.2)	
**Length of IOP^d^ stay, mean (SD); Welch *F_2,411.943_*=19.67, *P*<.001**								
	Average length of stay (in days)	53.88 (34.79)		74.67 (41.78)		54.67 (33.31)	58.10 (38.156)	
	Missing	N/A		N/A		N/A	0	
**Discharged against staff advice, n (%); *χ*^2^_2_=0.8, *P*=.67**								
	Yes	24 (9.4)		17 (8.9)		66 (10.8)	107 (10.1)	
	No	232 (90.6)		175 (91.1)		546 (89.2)	953 (89.9)	
	Missing	N/A		N/A		N/A	0	
**Used insurance for services, n (%): *χ*^2^_2_=2.0, *P*=.36**								
	Yes	247 (96.5)		185 (96.4)		599 (97.9)	1,031 (97.3)	
	Self-pay	9 (3.5)		7 (3.6)		13 (2.1)	29 (2.7)	
	Missing	N/A		N/A		N/A	0	
**Active SUD^e^ diagnosis, n (%)**								
	Alcohol use disorder; *χ*^2^_2_=0.2, *P*=.93	227 (88.7)		172 (89.6)		542 (88.6)	941 (88.8)	
	Cannabis use disorder; *χ*^2^_2_=0.3, *P*=.88	51 (19.9)		42 (21.9)		128 (20.9)	221 (20.8)	
	Cocaine use disorder; *χ*^2^_2_=5.0, *P*=.08	22 (8.6)		15 (7.8)		30 (4.9)	67 (6.3)	
	Hallucinogen use disorder; *χ*^2^_2_=1.5, *P*=.48	2 (0.8)		3 (1.6)		4 (0.7)	9 (0.8)	
	Inhalant use disorder; *χ*^2^_2_=4.5, *P*=.10	0		1 (0.5)		0	1 (0.1)	
	Opioid use disorder; *χ*^2^_2_=0.7, *P*=.71	19 (7.4)		17 (8.9)		56 (9.2)	92 (8.7)	
	Sedative use disorder; *χ*^2^_2_=0.3, *P*=.85	24 (9.4)		15 (7.8)		53 (8.7)	92 (8.7)	
	Other stimulant use disorder; *χ*^2^_2_=2.4, *P*=.30	31 (12.1)		15 (7.8)		59 (9.6)	105 (9.9)	
	Other psychoactive use disorder; *χ*^2^_2_=1.0, *P*=.61	4 (1.6)		4 (2.1)		7 (1.1)	15 (1.4)	
	Missing	N/A		N/A		N/A	0	
**Number of co-occurring SUD diagnoses^c^, n (%); *χ*^2^_6_=8.4, *P*=.21**								
	1	168 (65.6)		134 (69.8)		422 (69.0)	724 (68.3)	
	2	61 (23.8)		31 (16.1)		135 (22.1)	227 (21.4)	
	3	21 (8.2)		20 (10.4)		37 (6.0)	78 (7.4)	
	4	6 (2.3)		7 (3.6)		18 (2.9)	31 (2.9)	
	Missing	N/A		N/A		N/A	0	

^a^Clinical variables associated with a patient’s treatment measured included the patient’s length of IOP stay (in days), whether the patient was discharged against staff advice (yes/no), whether the patient used insurance or self-pay to finance their treatment (yes/no), the patient’s active SUD diagnoses (eg, alcohol, opioids), and the number of co-occurring SUD diagnoses.

^b^Variables where categories were collapsed into 2-4 levels in order to test for group differences due to small cell sizes.

^c^N/A: not applicable.

^d^IOP: intensive outpatient programming.

^e^SUD: substance use disorder.

**Table 2:** Sample characteristics of completers of the 3-month follow-up survey and noncompleters.^a^

Variables			Completers (N=1060)			Noncompleters (N=2503)			Statistics				
		n (%)		Mean (SD)	n (%)		Mean (SD)	*F* test (*df*)		*χ*^2^ (*df*)		*P* value	
**Demographic characteristics**													
	Biological sex: male	657 (62.0)		N/A^b^	1554 (62.1)		N/A	N/A		0.01 (1)		.93	
	Age (years)	1060 (100.0)		42.26 (12.93)	2503 (100.0)		38.26 (13.17)	69.35 (1,3561)		N/A		<.001	
	Race: White	973 (91.8)		N/A	2249 (89.8)		N/A	N/A		3.2 (1)		.07	
	Ethnicity: Latinx	51 (4.8)		N/A	135 (5.4)		N/A	N/A		0.6 (1)		.45	
**Employment**											16.1 (2)		<.001
	Full-time	672 (63.4)		N/A	1400 (55.9)		N/A	N/A		N/A		N/A	
	Unemployed	232 (21.9)		N/A	677 (27.0)		N/A	N/A		N/A		N/A	
**Education**											1.8 (2)		.40
	General Educational Development (GED) or less	116 (10.9)		N/A	306 (12.2)		N/A	N/A		N/A		N/A	
	Some college or bachelor’s degree	510 (48.1)		N/A	1180 (47.1)		N/A	N/A		N/A		N/A	
**Marital status**											50.6 (2)		<.001
	Married/life partner	550 (51.9)		N/A	985 (39.4)		N/A	N/A		N/A		N/A	
	Single	369 (34.8)		N/A	1160 (46.3)		N/A	N/A		N/A		N/A	
**Clinical characteristics**													
	Single active SUD^c^	724 (68.3)		N/A	1489 (59.5)		N/A	N/A		24.6 (1)		<.001	
	Discharged against staff advice	107 (10.1)		N/A	531 (21.2)		N/A	N/A		62.6 (1)		<.001	
	Length of stay	1060 (100.0)		58.10 (36.16)	2503 (100.0)		48.57 (38.73)	37.60 (1,3561)		N/A		<.001	
	Step down into IOP^d^	559 (52.7)		N/A	1259 (50.3)		N/A	N/A		1.8 (1)		.18	

^a^Mean (SD) reported for continuous variables and proportions (%) of samples reported for categorical variables. Pairwise differences calculated with chi-square tests and ANOVAs, as appropriate.

^b^N/A: not applicable.

^c^SUD: substance use disorder.

^d^IOP: intensive outpatient programming.

### Multivariate Comparisons

A few differences emerged between IOP settings across multiple domains of functioning (Table 3). There was no significant difference by setting in self-reported continuous abstinence, with over two-thirds of the sample (680/960, 70.8%) reporting no drug or alcohol use since discharge. Approximately one-third (332/1060, 31.3%) overall reported still being prescribed an anticraving medication. Of those, no difference in medication compliance emerged between in-person, hybrid, or virtual IOP respondents. Individuals across all settings reported attending peer support meetings, on average, of 1 or 2 times per week. Further, there were no differences across settings in the *overall* perceived quality of life or in the total number of poor physical and mental health days at 3-month follow-up. Finally, there were no differences detected between IOP setting and the individuals’ confidence in their ability to stay sober, financial well-being, or psychological well-being.

The only significant difference by IOP setting that emerged was in the overall quality of one’s *general health*, where those in the hybrid group (mean 4.08 [SD 0.75]) were more likely than those in the virtual group (mean 3.89 [SD 0.83]) to report a higher level of general health.

**Table 3:** Differences by IOP^a^ setting at 3-month follow-up.^b^

Variable	In-person (N=256)			Hybrid (N=192)			Virtual (N=612)			Statistics		
	n/N (%)	Mean (SD)	n/N (%)		Mean (SD)	n/N (%)		Mean (SD)	*F* test (*df*)		*χ*^2^ (*df*)	*P* value
Continuous abstinence	162/231 (70.1)	N/A^c^	131/180 (72.8)		N/A	387/549 (70.5)		N/A	N/A		0.4 (2)	.81
Craving medication compliance	58/75 (77.3)	N/A	46/55 (83.6)		N/A	154/202 (76.2)		N/A	N/A		1.4 (2)	.50
Peer support meeting attendance	235/256 (91.8)	2.54 (1.51)	180/192 (93.8)		2.56 (1.51)	567/612 (92.6)		2.60 (1.52)	0.35 (2,979)		N/A	.86
Overall quality of life	231/256 (90.2)	3.99 (0.83)	176/192 (91.7)		4.09 (0.74)	422/612 (69.0)		3.95 (0.81)	2.06 (2,826)		N/A	.13
Overall quality of general health	225/256 (87.9)	4.00 (0.84)	175/192 (91.1)		4.08 (0.75)	520/612 (85.0)		3.89 (0.83)	4.19 (2,917)		N/A	.01
Total number of poor physical and mental health days	224/256 (87.5)	3.70 (6.66)	176/192 (91.7)		3.59 (6.50)	531/612 (86.8)		3.83 (6.43)	0.10 (2,928)		N/A	.90
Self-efficacy for staying sober	220/256 (85.9)	5.93 (1.16)	173/192 (90.1)		6.00 (1.06)	551/612 (90.0)		5.95 (1.27)	0.21 (2,941)		N/A	.81
Psychological well-being	219/256 (85.5)	45.09 (9.40)	170/192 (88.5)		45.83 (7.51)	532/612 (86.9)		44.91 (8.86)	0.72 (2,918)		N/A	.49
Financial well-being	220/256 (85.9)	48.40 (8.52)	170/192 (88.5)		46.73 (7.96)	380/612 (62.1)		47.20 (8.16)	2.30 (2,767)		N/A	.10

^a^IOP: intensive outpatient programming.

^b^Mean (SD) reported for continuous variables and proportions (%) of samples reported for categorical variables. Pairwise differences calculated with chi-square tests and ANOVAs, as appropriate.

^c^N/A: not applicable.

## Discussion

### Principal Findings

This study is the first of its kind to assess telehealth for SUD in the IOP setting in a large cohort of patients (N=1000+). No meaningful differences in outcome measures were identified between delivery settings at 3-month follow-up, with individuals reporting similar levels of continuous abstinence, quality of life, and social/emotional well-being. Our findings in regard to continuous abstinence were consistent with previous studies following patients at 3-6 months postdischarge from IOP (eg, 65/103 [63.1%]) [33]. These results are promising and suggest a potential continuing role for virtual IOP as an effective component in addiction treatment settings. Advocacy is needed to maintain these services as a standard offering within the SUD treatment continuum.

Historically, peer-based connections and the therapeutic milieu have been integral parts of addiction treatment. Concern has been expressed by the addiction treatment community regarding the shift to virtual services and its impact on group engagement and patient-centered outcomes [34]. These preliminary results demonstrate the feasibility of offering services virtually. Further research is necessary to obtain feedback on patient experience and measures of group cohesion, such as secure emotional expression, as they apply to virtual addiction treatment [35].

Our findings aid in establishing a platform for future evaluation of data collection processes that inform the effective development of standardized protocols for routine outcomes data practices, including frequency of contact, method of outreach, and training of staff. Standardized protocols must consider the context for accurate interpretation of collected data. For example, differences in response rates emerged in this study based on the timing of data collection in relation to the global pandemic and due to unanticipated staff burden and should be interpreted in this context. At 3-month follow-up, response rates were lowest for those in in-person IOP (256/957, 26.8%). These rates are likely attributable to these being completed by patients between May and August 2020, timing that coincided with major city- and statewide lockdowns and great uncertainty about the unknowns presented by the pandemic. Furthermore, because of the large opt-in of the in-person cohort at the beginning of data collection (May 2020), many participants at 3-month follow-up were given access to the survey well into the 30-day response window, reducing the likelihood that they would have adequate time to complete it prior to survey close. In contrast, response rates for those in virtual IOP (612/1532, 28.5%) were most impacted by a higher-than-projected admission rate of individuals into IOP throughout the study period, resulting in a larger sample size than originally anticipated. As a result, there were proportionally lower staffing levels than would be typically allocated for the final sample size, which may explain the lower response rates for the virtual group.

### Strengths and Limitations

A key strength of this study is the breadth of data collected from such a large number of patients receiving SUD treatment during a period of extensive change. Although these results are mainly descriptive, these analyses are necessary to carefully evaluate the impacts of a global shift in treatment approach. A number of limitations should be considered when interpreting results. Similar to existing research used with ROM data [36-39], response rates at 3-month follow-up were low. Nonresponse bias is a risk inherent to survey analysis. However, even in studies with high response rates, research has shown that nonresponse rates are not always directly predictive of nonresponse bias [37,40-42]. Research supports that highly resource-intensive recruitment deployed to capture late responders does not necessarily alter the outcomes found at lower, less resource-intense response rates, and this type of recruitment can be both cost- and time-prohibitive [40,42]. Despite COVID-induced high nonresponse rates, we expect these data to accurately reflect the effectiveness of IOP services during the COVID-19 pandemic. Substance use at follow-up was based on retrospective self-report of use. Utilizing additional methods for verification, such as urine drug analyses, would strengthen the validity of these reports in future studies. As discussed earlier, due to the sudden onset of the pandemic and subsequent data collection for this unique cohort, baseline and 1-month follow-up data were unable to be collected from a substantial portion of the full sample. The missing data influenced our ability to directly compare the effectiveness of IOP across delivery type, given the inherent confounding effect of the timing of patient care in relation to the unfolding public health crisis. We recommend future prospective studies be designed to compare in-person and virtual treatment directly, with inclusion of a formal evaluation of the ideal conditions for patient success (ie, dosage, treatment duration, frequency).

Finally, although our sample was representative of HBFF program participants, it differs from the general population in a few specific ways that are important to acknowledge when considering the generalizability of the findings. First, patients in the sample were primarily White and non-Latinx. As a result, there may have been too few non-White/Latinx participants to detect differences. However, this sample directly compares to findings from the 2020 National Survey on Drug Use and Health (NSDUH) [43] in terms of full- and part-time employment (United States: 67.3% vs sample: 67.0%) and bachelor’s degree attainment (United States: 27.7% vs sample: 29%) among adults 18+ years old with a SUD in the past year. This demonstrates that addiction affects individuals across varying educational and employment statuses. This is in contrast to stereotypes of individuals struggling with addiction, which may characterize this as a disease of the uneducated and unemployed. Even though a higher percentage of patients employed full-time completed the 3-month survey (672/1060 [63.4%] vs 1400/2503 [55.9%]), post hoc analyses revealed no difference in outcomes based on employment status; therefore, we believe this had minimal impact on our findings. Future research should endeavor to improve the representation of racial and ethnic minorities in order to improve generalizability across a wider cross section of demographic variables.

### Conclusions

Results from this study suggest that virtual outpatient care for the treatment of SUDs is a feasible alternative to in-person care, leading to similar rates of self-reported continuous abstinence, health, and well-being in patients at 3-month follow-up. This study should serve as a baseline for the assessment and refinement of the role of virtual services in the field of addiction treatment in order to better understand under what circumstances telehealth can function as an effective alternative to the established in-person standard of care.

## Abbreviations

AA: Alcoholics Anonymous
CFPB: Consumer Financial Protection Bureau
HBFF: Hazelden Betty Ford Foundation
IOP: intensive outpatient programming
RDCS: research data collection specialists
ROM: routine outcome monitoring
SUD: substance use disorder
